# PROTOCOL: Non‐pharmacological interventions for older people with a diagnosis of depression: An evidence and gap map

**DOI:** 10.1002/cl2.1354

**Published:** 2023-09-27

**Authors:** Wenru Shang, Liping Guo, Yujia Liu, Yanfei Li, Qian Wei, Ke Guo, Minyan Yang, Lili Wei, Zheng Xu, Junqiang Niu, Xiuxia Li, Kehu Yang

**Affiliations:** ^1^ School of Basic Medical Sciences, Evidence‐Based Medicine Center Lanzhou University Lanzhou China; ^2^ Collaborative Innovation Center of First Hospital of Lanzhou University Lanzhou China; ^3^ School of Public Health, Evidence‐Based Social Science Research Center Lanzhou University Lanzhou China; ^4^ Gansu University of Traditional Chinese Medicine Lanzhou China; ^5^ Key Laboratory of Public Health Safety, Ministry of Education, School of Public Health Fudan University Shanghai China; ^6^ Lanzhou University First Affiliated Hospital Lanzhou China

## Abstract

This is the protocol for an evidence and gap map. The objectives are as follows: To map available randomized control trials, economic evaluations, and systematic reviews that assess the effectiveness and cost‐effectiveness of non‐pharmacological interventions for older people with a diagnosis of depression and identify any existing gaps in the evidence that can inform future research.

## 1 BACKGROUND

### 1.1 Introduction

The significant rise in age‐related illnesses among an increasingly aging population presents one of the primary challenges facing modern society (Christensen, 2009; Partridge, 2018). The process of aging is often coupled with declining immune function and the onset of various diseases, as well as with low material and spiritual living standards. The resulting problems can render older adults more vulnerable to experiencing psychological problems such as fear, loneliness, and depression (Blazer, 2020). Of these, depression is the most common mental health problem in older adults (Han, 2018; Jonsson, 2016; Porensky, 2009; Wilkinson, 2018). Estimates have indicated that nearly 14% of people over the age of 55 suffer from depression (Kok, 2017; Vink, 2009). Additionally, the economic costs of depression are substantial, encompassing not only the direct costs of treatment, but also the indirect costs associated with premature mortality and morbidity (Katon, 2003; Vos, 2020). A review has shown that the estimated total cost of services to treat depression in the UK in 2007 was a staggering £1.7 billion; if the cost of lost employment is also factored in, the total cost increases to £7.5 billion. By 2026, these figures have been projected to increase to £3 billion and £12.2 billion, respectively (McCrone, 2008).

According to the Diagnostic and Statistical Manual of Mental Disorders, Fifth Edition (DSM‐5), a Major Depression Episode (MDE) diagnosis requires five or more symptoms to be present within a 2‐week period (APA, 2013). One of these symptoms can be either a depressed mood or anhedonia (loss of interest or pleasure, LI). The secondary symptoms of MDE include appetite or weight changes, sleep difficulties (insomnia or hypersomnia), psychomotor agitation or retardation, fatigue or loss of energy, diminished ability to think or concentrate, feelings of worthlessness or excessive guilt, and suicidality. These symptoms are commonly rated in an all or none (0 or 1) fashion (APA, 2013). Notably, the clinical symptoms of depression differ between older adults and younger adults. Depressive mood and loss of interest are recognized as core symptoms of depression in both older and younger adults. However, older patients may experience additional core symptoms of depression such as death wishes and pessimism, whereas younger individuals may predominantly experience symptoms such as fatigue and changes in appetite (Baba, 2022; Fried, 2016). Moreover, studies have shown that with increasing age, the course of depression worsens (Mirza, 2016; Schaakxs, 2018).

Treatments for depression can be broadly categorized into pharmacological interventions, non‐pharmacological interventions, or a combination of both (NICE, 2009). Pharmacological treatments include a range of antidepressant drugs, including selective serotonin inhibitors and selective norepinephrine reuptake inhibitors (Furukawa, 2016). In clinical practice, prescribing antidepressant medications is the primary approach to managing depression in most patients. However, because depression in older adults often co‐occurs with non‐communicable diseases, such as cardiovascular disease, diabetes, cancer, and chronic respiratory diseases, concerns regarding polypharmacy have been raised (Gunn, 2012; Holvast, 2017). Furthermore, clinical studies have shown that prescribing antidepressants to older patients increases their risk of adverse drug‐related events and potential drug interactions with other medications (Everitt, 2018; Landi, 2007; Tham, 2016). For instance, Sobieraj et al. found a strong correlation between the increasing trend that older adults take multiple medications and the heightened risk of adverse outcomes, including falls and cognitive impairment (Sobieraj, 2019).

Non‐pharmacological interventions for depression include a range of approaches, such as psychotherapy (e.g., reminiscence therapy, mindfulness‐based cognitive therapy, and music therapy) and non‐invasive brain stimulation therapies (e.g., modified electroconvulsive therapy and repetitive transcranial magnetic stimulation) (Gertler, 2015). Evidence suggests that these non‐pharmacological interventions are effective in relieving the symptoms of depression among older adults (Apóstolo, 2016; Baba, 2022; Chan, 2021; Hall, 2016; Holvast, 2017). For instance, the Japanese Agency for Medical Research and Development (AMED) Guideline Development Panel has recommended several psychotherapies as effective interventions for reducing depressive symptoms in older adults. These include cognitive‐behavioral therapy (CBT), problem‐solving therapy, reminiscence therapy/life review therapy, and behavioral activation therapy. These therapies have been found to be effective without being associated with any notable adverse events (Baba, 2022; Frost, 2019; Gould, 2012). Considering the potential limitations and risks associated with pharmacological treatments in older adults, these findings have sparked increasing interest for non‐pharmacological interventions for depression, particularly in this specific population.

### 1.2 Interventions

Non‐pharmacological interventions for depression are manifold (Gertler, 2015). These interventions are primarily psychological in nature but also include medical, physical, and other interventions.

- Based on the Clinical Guidelines for the treatment and management of depression in adults issued by the National Institute for Health and Clinical Excellence (NICE) (NICE, 2009), as well as the comprehensive list of 87 psychological interventions provided by the Cochrane Common Mental Disorders Group (formerly the Depression, Anxiety, and Neurosis Group) (CCDAN, 2013), we have identified a set of 17 widely recognized and evidence‐based psychological interventions. These interventions include acceptance and commitment therapy (ACT), animal‐assisted therapy, art therapy, behavioral therapy (BT), cognitive behavioral therapy (CBT), cognitive bias modification (CBM), computer‐assisted therapies (professionally guided), dance and movement therapy (DMT), dialectical behavior therapy (DBT), emotion‐focused therapy (EFT), metacognitive therapy (MCT), problem‐solving therapy (PST), psychodynamic therapies, solution‐focused therapy (SFT), humanistic therapies, mindfulness‐based cognitive therapy (MBCT), and family therapy.
- Non‐pharmacological medical interventions include electroconvulsive therapy (ECT), repetitive transcranial magnetic stimulation (rTMS), neurosurgical interventions, and biofeedback.
- Physical interventions include exercise programs and other physical activation strategies.
- Complementary and alternative medicine (CAM) interventions have also been described, which include St. John's wort, Tryptophan/5‐Hydroxytryptophan, S‐adenosyl methionine, folate, inositol, acupuncture, saffron (herbal medicine), complex homeopathy, and relaxation training (Thachil, 2007).

### 1.3 Why it is important to develop an evidence and gap map (EGM)?

Several reviews of non‐pharmacological treatments for older people with a diagnosis of depression have been published, indicating a notable increase in research in this area (Apóstolo, 2016; Chen, 2021; Holvast, 2017; Krause, 2019). This growing focus can be attributed to the expanding aging population and its associated needs. However, the obtained findings regarding the effectiveness of such interventions have not always been consistent (Baba, 2022). The rapid expansion of evidence in support of their use, particularly in the absence of a comprehensive overview of existing evidence, can lead to the duplication of studies and a lack of clarity in research direction.

An EGM serves as a valuable decision‐making and research prioritization tool. It helps to highlight research gaps, thus enabling researchers and decision‐makers to make informed decisions based on available evidence. EGMs support the creation of evidence‐informed policies and help to guide research prioritization (Snilstveit, 2016). EGMs also help to avoid unnecessary duplication and to assess the sufficiency of evidence for the synthesis of knowledge and for decision‐making (Snilstveit, 2016; White, 2020). Therefore, the objective of this study is to generate an EGM to provide a comprehensive overview of the existing evidence and identify knowledge gaps concerning the effectiveness of non‐pharmacological interventions for depression in older adults.

### 1.4 Existing EGMs

We conducted searches in the Campbell Library, Cochrane Library, PubMed, Evidence for Policy and Practice Information (EPPI), and International Initiative for Impact Evaluation (3ie) databases, resulting in the identification of one relevant EGM pertaining to non‐pharmacological interventions of depression. Farah et al. (2016) constructed an evidence map based on original studies and found that the quality of evidence was predominantly low across most comparisons (Farah, 2016). However, this EGM analyzed evidence across all populations with depression rather than specifically focusing on older individuals with depression. Additionally, the process of creating the EGM remains unclear because of a lack of description regarding inclusion and exclusion criteria, analysis methods, and other relevant details. The EGM developed in the present paper on non‐pharmacological interventions for older people with a diagnosis of depression adheres to the Campbell Guidelines for EGMs (White, 2020). This work provides valuable insights for both researchers and decision‐makers, thus informing future investigations in this area and supporting the improvement of health and social care practices.

## 2 OBJECTIVES

The objectives of this EGM are to:

- Map available randomized control trials (RCT), economic evaluations, and systematic reviews that assess the effectiveness and cost‐effectiveness of non‐pharmacological interventions for older people with a diagnosis of depression.
- Identify any existing gaps in the evidence that can inform future research.

## 3 METHODS

### 3.1 EGM: Definition and purpose

EGMs offer a visual representation of evidence derived from systematic reviews and impact evaluations of a specific topic, theme, or sector, organized around a framework (matrix) of key interventions and outcomes (White, 2020). EGMs generally have a broader scope compared to systematic reviews. They typically include systematic reviews and primary studies, but may include only one of these, and may sometimes also include other maps (White, 2020). As stated by White (2020), the main uses of EGMs are to: (1) guide users toward available relevant evidence to inform the design and implementation of interventions; (2) identify existing high‐quality reviews as a basis for evidence summaries for policy purposes or to populate evidence portals; (3) inform implementing agencies if there is no relevant evidence for their interventions; and (4) identify research gaps for new primary research and new syntheses.

Based on this concept, our EGM will provide a visual overview of the available evidence on the effectiveness of non‐pharmacological interventions for older individuals diagnosed with depression. The EGM indexes systematic reviews and primary studies on effectiveness and cost‐effectiveness to identify existing knowledge about non‐pharmacological interventions. Furthermore, this EGM will present the available evidence in a graphical matrix, where interventions are listed in rows and indicators/outcomes are listed in columns, showcasing areas that are characterized by strong, weak, or nonexistent evidence. We will incorporate EGM methods from relevant studies and employ the following five‐stage process:

- Establish a framework that defines the scope and sets inclusion and exclusion criteria.
- Identify available evidence through a comprehensive search.
- Assess the quality of the evidence through a rigorous appraisal process.
- Extract, code, and summarize data relevant to objectives.
- Present findings in a user‐friendly format, utilizing visualization techniques.

We will employ the EPPI‐Mapper mapping tool (EPPI‐Mapper 2021), developed by the Evidence for Policy and Practice Information (EPPI) and Co‐ordinating Centre, to visually present the identified studies within the framework described below.

### 3.2 Framework development and scope

After engaging in discussions and receiving recommendations from stakeholders and advisory panels on March 10, 2023, a framework for the primary classification of items pertaining to various intervention populations and groups was finalized. We will further define the scope and framework in consultation with our research group, which consists of experts from various fields. The framework used in the EGM will initially follow the traditional intervention‐outcome matrix, with rows containing non‐pharmacological intervention domains and columns containing depression outcome categories. Both interventions and outcomes will be extended with relevant subcategories if necessary. In addition, a framework of specific interventions and outcomes will be developed through an integrative framework and a detailed review of psychosocial theory (Kinser, 2014) as well as research on depression (Dirmaier, 2012; Gertler, 2015; Guo, 2023; Sikkes, 2021).

### 3.3 Stakeholder engagement

During the development of this EGM, we will engage with a wide range of stakeholders to gather valuable input. These stakeholders will include evaluation and evidence synthesis (LYF, GLP, and YKH), public health (LMX, WQ, and LXX), psychiatry (NJQ), psychotherapy (LYJ and XZ), as well as neurological patients and members of the public. Stakeholders in this EGM are mainly from the following organizations:

- The Center for Evidence‐based Medicine and Evidence‐based Social Science Research (evidence synthesis: YKH, LYF, and GLP).
- First Hospital of Lanzhou University (psychiatry: NJQ; neurological patients).
- Gansu University of Traditional Chinese Medicine (public health: LLW; psychotherapy: LYJ).
- Key Laboratory of Evidence‐Based Medicine and Knowledge Translation (public health: LMX and LXX; psychotherapy: XZ).
- Key Laboratory of Public Health Safety, Ministry of Education (public health: WQ).

Stakeholders will actively engage in every stage of the EGM process and will provide valuable input and feedback on interventions, studies, outputs, mapping findings, and dissemination channels. While all stakeholders play an important role, it is worth noting that patients and the public are primarily involved in discussions associated with the conceptual framework; moreover, patient information will be kept confidential and will not be released. Initial discussions will focus on defining and refining research questions and protocols, including the development of a coding framework based on selected studies that were either included or excluded. Subsequent consultations will then shift toward the actual production of the EGM.

### 3.4 Conceptual framework

**Figure 1:**
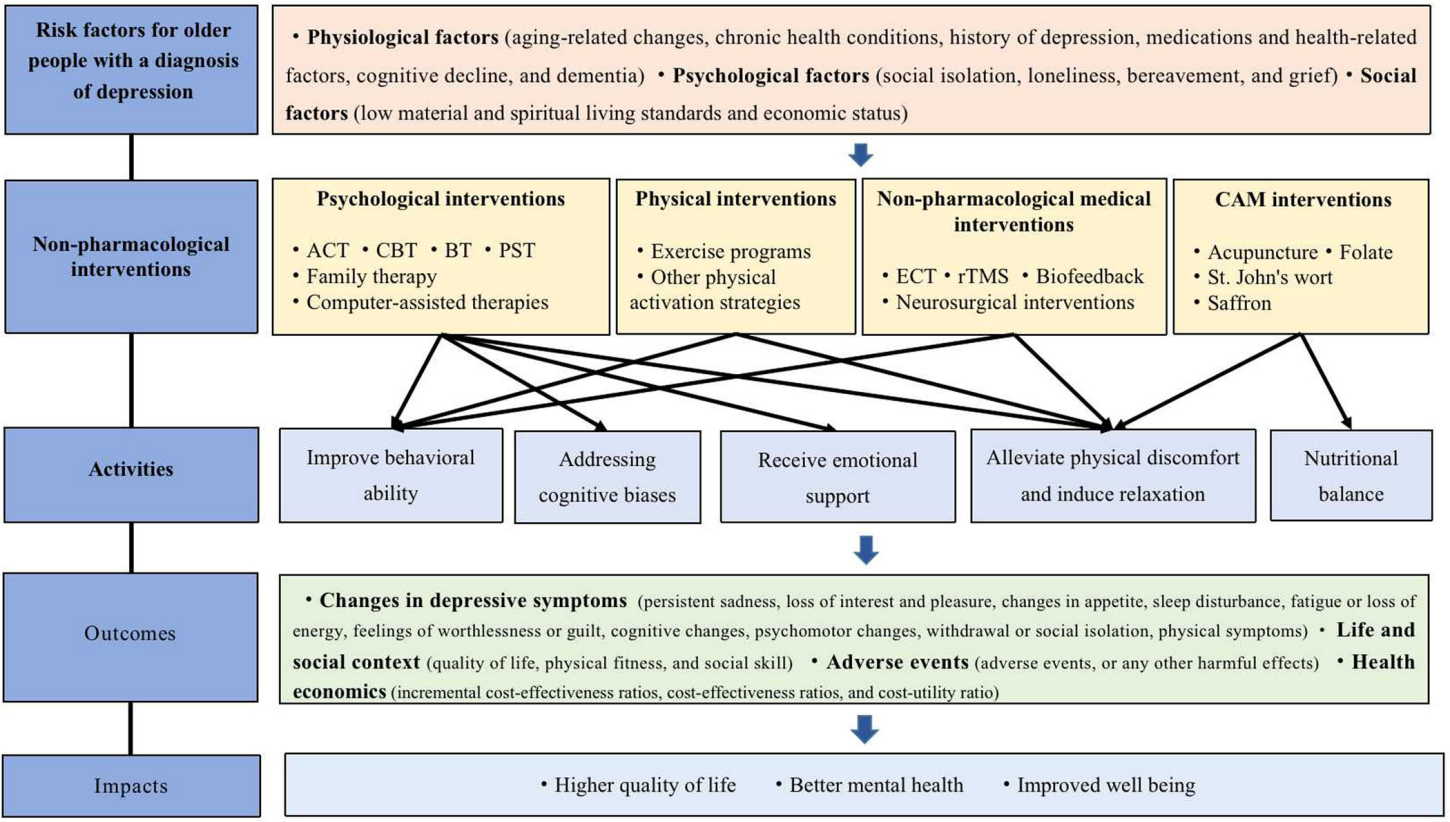
The conceptual framework for older people with a diagnosis of depression. ACT, acceptance and commitment therapy; BT, behavioral therapy; CBT, cognitive behavioral therapy; ECT, electroconvulsive therapy; PST, problem‐solving therapy; rTMS, repetitive transcranial magnetic stimulation.

Kinser et al. (2014) introduced a framework that explores the intricate and bidirectional relationship between vulnerability to stress, depression, and health outcomes in women. This framework has been utilized in research on complementary therapies for depression (Kinser, 2014). Sikkes et al. (2021) developed a theory‐based specification of non‐pharmacological treatments of aging and dementia. This specification includes treatment targets, which are defined as the functional aspects of clinicians aim to modify (e.g., improved semantic recall, or increased adherence to an exercise regimen), as well as ingredients, which are defined as actions of clinicians or objects hypothesized to bring about the desired change (e.g., instructions, modeling, and coaching) (Sikkes, 2021). Additionally, Guo et al. presented a conceptual framework for depression in their EGM protocol for the treatment of depressive disorders among adults, which includes risk factors, intervention, activities, outcomes, and impacts (Guo, 2023). Our conceptual framework is based on the existing and applied frameworks mentioned above. It incorporates elements such as risk factors for older people with a diagnosis of depression, non‐pharmacological interventions, activities, outcomes, and impacts (Figure 1).

Intervention categories are based on the four categories of non‐pharmacological intervention: psychological interventions, non‐pharmacological medical interventions, physical interventions, and complementary and alternative medicine (CAM) interventions (Gertler, 2015). Health outcomes include various aspects, such as a change in depressive symptoms, relief from anxiety, easing of stress, reduction of suicidal ideation, improvement in the quality of life, enhancement of physical fitness, and increase in social skills (Guo, 2023). In addition, health economic outcomes include cost‐effectiveness ratios, cost‐utility ratios, and incremental cost‐effectiveness ratios of different non‐pharmacological interventions.

## 4 DIMENSIONS

As stated above, this EGM will be presented as a matrix of interventions (rows) and outcomes (columns). The following interventions and outcomes will be refined after consultation with stakeholders.

### 4.1 EGM framework: Interventions

Table 1 lists the intervention categories, examples of subcategories, and definitions. The included interventions cover all non‐pharmacological interventions across psychological interventions, non‐pharmacological medical interventions, physical interventions, and CAM interventions.

**Table 1:** Categories, subcategories, definitions, and examples of interventions.

Categories	Sub‐category	Definition	Examples
Psychological interventions	Acceptance and commitment therapy (ACT)	*ACT is a type of psychotherapy that focuses on helping individuals develop psychological flexibility by accepting their thoughts and emotions while committing to taking meaningful action aligned with their values* (Hayes, 2012)	“Therefore, the current research aimed to determine the effectiveness of acceptance and commitment‐based therapy on depression, psychological health and life expectancy of the elderly with nonclinical depression.” (Golestanifar, 2020)
	Animal‐assisted therapy	Animal‐assisted therapy is a therapeutic intervention that incorporates animals, such as horses, dogs, cats, and birds, into the treatment plan (Aubrey, 2019)	“The aim of this study was to verify dog‐assisted therapy's effectiveness on depression and anxiety in institutionalized elderly.” (Ambrosi, 2019)
	Art therapy	Art therapy involves the use of creative techniques such as drawing, painting, collage, coloring, or sculpting to help people express themselves artistically and examine the psychological and emotional undertones in their art (Case, 2014)	“This study aimed to systematically review and meta‐analysis of clinical trials, summarize eligible relevant studies and provide a true effect measure for the association between AT and depression symptoms in older adults.” (Jenabi, 2023)
	Behavioral therapy (BT)	*BT is a form of psychotherapy that focuses on understanding and modifying behaviors that are causing distress or contributing to psychological difficulties. It is based on the principle that our behaviors are learned and can be changed through targeted interventions* (Wilson, 2005)	
	Cognitive behavioral therapy (CBT)	*CBT is a widely recognized and evidence‐based therapeutic approach that focuses on the connection between thoughts, feelings, and behaviors. It aims to help individuals identify and change negative patterns of thinking and behavior that contribute to their distress or mental health difficulties* (Rothbaum, 2000)	“To review the effectiveness of cognitive behavioral therapy (CBT) for depression in older people, together with factors associated with its efficacy.” (Gould, 2012)
	Cognitive bias modification (CBM)	*CBM is an approach that targets and modifies specific cognitive biases, which are automatic and unconscious patterns of thinking that can contribute to negative emotions and problematic behaviors. It is based on the understanding that biased information processing can play a role in the development and maintenance of various psychological disorders* (Koster, 2009)	“This study aimed to elucidate the effect of cognitive bias modification on depression.” (Li, 2023)
	Computer‐assisted therapies (professionally‐guided)	*Computer‐assisted therapies refer to therapeutic interventions that incorporate the use of technology, specifically computers, to support and enhance the therapeutic process. These therapies are typically guided by a trained professional, such as a therapist or counselor, who utilizes computer‐based tools and programs as part of the treatment* (Carroll, 2010)	“To evaluate the efficacy of computer‐assisted forms of cognitive‐behavior therapy for major depressive disorder (MDD) and examine the role of clinician support and other factors that might affect outcomes.” (Wright, 2019)
	Dance and movement therapy (DMT)	*DMT is a form of expressive therapy that utilizes movement, dance, and body awareness as a means of promoting emotional, cognitive, physical, and social well‐being. It is based on the belief that the body and mind are interconnected, and that engaging in expressive movement can facilitate personal growth and healing* (Payne, 2003).	“This systematic review assessed the published literature on dance movement therapy interventions with adults aged 60 years and older with a mental health disorder.” (Jiménez, 2019)
	Dialectical behavior therapy (DBT)	*DBT is a form of CBT that utilizes both behavioral and cognitive techniques to help people learn to manage their emotions, cope with distress, and improve interpersonal relationships* (Swales, 2009).	“Results from these two treatment development studies indicate that applying standard DBT for the treatment of co‐morbid MDD or MDD + PD (personality disorders) in older adults is feasible, acceptable, and has clinical promise.” (Lynch, 2007)
	Emotion‐focused therapy (EFT)	*EFT is an evidence‐based therapeutic approach that focuses on the role of emotions in psychological well‐being and the therapeutic process. EFT is grounded in the belief that emotions are adaptive and that working with and understanding emotions can lead to personal growth and healing* (Greenberg, 2004).	“This study aims to examine a transdiagnostic adaptation of EFT (EFT‐T) as a treatment for depression, anxiety and related disorders” (Timulak, 2020)
	Metacognitive therapy (MCT)	*MCT is a form of psychotherapy that focuses on targeting and modifying unhelpful thinking patterns and metacognitive processes. MCT is based on the metacognitive model of psychological disorders, which suggests that it is not the content of our thoughts but rather our thinking processes and beliefs about thoughts that contribute to emotional distress and psychological difficulties* (Wells, 2011).	“The aim of this study was to compare the effectiveness of metacognitive therapy and emotion efficacy therapy on the level of depression and self‐care ability in non‐clinical depressed elderly.” (Abdi, 2020)
	Problem‐solving therapy (PST)	*PST is a brief, structured, and goal‐oriented form of psychotherapy that focuses on developing practical skills to effectively address and solve specific problems. It is based on the belief that enhancing problem‐solving abilities can lead to improved coping, increased self‐efficacy, and better overall well‐being* (D'Zurilla, 2010).	“We examined the effectiveness of PST for the treatment of MDD in older adults in a systematic review and meta‐analysis.” (Kirkham, 2016)
	Psychodynamic therapies	*Psychodynamic therapies encompass a range of therapeutic approaches that aim to explore and understand the unconscious processes, conflicts, and patterns of behavior that contribute to psychological distress and interpersonal difficulties. These therapies draw from psychoanalytic theory and emphasize the role of early childhood experiences, unconscious motivations, and the therapeutic relationship in promoting insight, healing, and personal growth* (Huprich, 2009).	“To examine the effectiveness and acceptability of all psychodynamic therapy approaches compared with all other psychological therapy approaches for acute depression.” (Churchill, 2010)
	Solution‐focused therapy (SFT)	*SFT also known as Solution‐Focused Brief Therapy (SFBT), is a goal‐oriented therapeutic approach that focuses on identifying and building on an individual's strengths and resources to create positive change. SFT is based on the belief that individuals have the ability to create solutions to their problems and that the focus should be on finding what works rather than dwelling on the problems themselves* (Macdonald, 2014).	“This study aimed to investigate the effectiveness of solution‐focused group counseling (SFGC) to reduce depressive symptoms and improve cognitive functions among Chinese rural older adults.” (Wang, 2022)
	Humanistic therapies	*Humanistic therapies are a category of therapeutic approaches that emphasize the individual's unique experience, personal growth, and self‐actualization. These therapies focus on the person as a whole and aim to facilitate self‐awareness, self‐acceptance, and the development of one's full potential. Humanistic therapies are rooted in the belief that individuals have the inherent capacity for growth, self‐direction, and meaningful relationships* (Grogan, 2013).	“To examine the effectiveness and acceptability of all humanistic therapies compared with different psychological therapy approaches (psychodynamic, behavioural, humanistic, integrative, cognitive‐behavioural) for acute depression.” (Churchill, 2010)
	Mindfulness‐based cognitive therapy (MBCT)	*MBCT is an evidence‐based therapeutic approach that combines elements of cognitive therapy with mindfulness practices. It was specifically developed to help individuals who experience recurring episodes of depression or chronic unhappiness. MBCT aims to prevent relapse by teaching individuals to become more aware of their thoughts and emotions, and to relate to them in a non‐judgmental and accepting way* (Sipe, 2012).	“To review the evidence base for mindfulness‐based cognitive therapy for the treatment of anxiety and depression in older people.” (Thomas, 2020)
	Family therapy	*Family therapy, also known as family counseling or systemic therapy, is a type of therapeutic approach that focuses on understanding and addressing the dynamics and interactions within a family system. It recognizes that individuals are influenced by their family context and that changes in the family system can have a significant impact on individual well‐being* (Goldenberg, 2012).	“The purpose of this paper was to review the characteristics and findings of dyadic and family‐oriented interventions for late‐life mood disorders to determine if they are effective and beneficial.” (Stahl, 2016)
Non‐pharmacological medical interventions	Electroconvulsive therapy (ECT)	*ECT is a medical procedure used to treat certain mental health conditions, primarily severe depression, bipolar disorder, and sometimes schizophrenia. It involves the application of electric currents to the brain, which intentionally induces a controlled seizure. While the exact mechanisms of action are not fully understood, ECT is believed to influence neurotransmitter levels and promote changes in brain chemistry, leading to symptom improvement* (Espinoza, 2022).	“This study assessed whether inflammatory markers prior to ECT are associated with cognitive functioning in depressed patients treated with ECT.” (Carlier, 2021)
	Repetitive transcranial magnetic stimulation (rTMS)	*rTMS is a non‐invasive therapeutic technique used to modulate brain activity by applying magnetic fields to specific regions of the brain. It is primarily used in the treatment of certain mental health conditions, particularly major depressive disorder, but has also shown potential in other psychiatric and neurological conditions* (Lefaucheur, 2020).	“This study aims to systematically review and meta‐analyze evidence of rTMS efficacy in MDD treatment among older adults.” (Valiengo, 2022)
	Neurosurgical interventions	*Neurosurgical interventions refer to surgical procedures performed on the brain, spinal cord, or peripheral nervous system to treat a variety of neurological conditions. These interventions are typically conducted by neurosurgeons, who specialize in the surgical management of disorders affecting the nervous system. Neurosurgical interventions can involve different techniques and approaches depending on the specific condition being treated* (Yudofsky, 2008).	
	Biofeedback	*Biofeedback is a technique that enables individuals to gain awareness and control over certain physiological processes within their bodies. It involves using specialized equipment to measure and provide real‐time feedback about physiological functions such as heart rate, blood pressure, muscle tension, skin temperature, and brainwave activity* (Swingle, 2008).	
Physical interventions	Exercise programs	*Exercise programs, also known as fitness programs or workout routines, are structured plans designed to promote physical activity and improve overall fitness and health. These programs typically consist of a combination of various exercises and activities aimed at targeting specific fitness goals or addressing specific health needs* (Oeland, 2010).	“This study attempted to show evidence of exercise programs as intervention to decrease depressive symptoms and to improve quality of life and self‐esteem in older people.” (Park, 2014)
	Other physical activation strategies	*Physical activation strategiesare techniques or approaches that aim to increase physical activity levels and promote a more active lifestyle. These strategies can be useful for individuals of all ages and fitness levels* (Martinsen, 2008).	“This study examined the effect of a DVD‐delivered exercise intervention on the secondary outcomes of depression and anxiety in older adults and the extent to which physical self‐worth mediated the relationship between leisure‐time physical activity and depression and anxiety.” (Aguiñaga, 2018)
Complementary and alternative medicine interventions	St. John's wort (*Hypericum perforatum*)	*St. John's wort (Hypericum perforatum) is a herbaceous plant with yellow flowers that has been used for centuries as a herbal remedy. St. John's wort has a long history of traditional use for various medicinal purposes, particularly for its potential antidepressant properties* (Linde, 2005).	We found Grade 1 evidence for the use of *Hypericum perforatum* (St. John's Wort) in depressive disorders (Thachil, 2007).
	Tryptophan/5‐Hydroxytryptophan	*Tryptophan and 5‐HTP show promise in some studies, the evidence supporting their effectiveness as standalone treatments for mood disorders is limited and mixed. They are often used as complementary therapies alongside other treatments and medications* (Shaw, 2002).	We found Grade 1 evidence indicating some benefit from Nutritional Therapy in Depression, with Tryptophan/5‐Hydroxytryptophan (Thachil, 2007).
	S‐adenosyl methionine	*S‐adenosyl methionine (SAMe) is a naturally occurring compound in the body that plays a vital role in various biochemical processes. SAMe is commonly used as a dietary supplement for managing symptoms of depression* (Galizia, 2016).	We also found Grade 1 evidence for another Nutritional Therapy approach, with S‐adenosyl methionine (SAMe), indicating that it may be of some benefit in Depression (Thachil, 2007).
	Folate	*Folate, also known as vitamin B9, has been studied in relation to depression due to its involvement in the synthesis of A systematic review on the effectiveness of folate in the treatment of depression found only one trial (out of the 3 neurotransmitters, such as serotonin, dopamine, and norepinephrine, which play important roles in mood regulation* (Bender, 2017).	A systematic review on the effectiveness of folate in the treatment of depression found only one trial (out of the 3 included), which evaluated the use of folate as monotherapy (Thachil, 2007).
	Inositol	*Inositol is a naturally occurring sugar alcohol that is considered a pseudovitamin. Some studies have suggested that inositol supplementation may help alleviate symptoms of panic disorder, obsessivecompulsive disorder (OCD), and depression* (Mukai, 2014).	A systematic review to determine the effectiveness of inositol in treating depression identified four trials, with a total of 141 participants (Thachil, 2007).
	Acupuncture	*Acupuncture is a key component of Traditional Chinese Medicine (TCM) that has been practiced for thousands of years* (Smith, 2018).	This systematic review and meta‐analysis examined the effectiveness of acupuncture in major depressive disorder (Armour, 2019).
	Saffron (Herbal medicine)	*Saffron is a spice derived from the flower of Crocus sativus, commonly known as the saffron crocus. Some studies have indicated that saffron may be as effective as certain antidepressant medications in reducing symptoms of depression, particularly in individuals with mild to moderate depression* (Lopresti, 2014).	Two well‐designed RCTs from the same research group compared the effectiveness of the herb Saffron (*Crocus sativus* L.) against standard antidepressants in mild to moderate depression, diagnosed according to the Structured Clinical Interview for DSM‐IV (Thachil, 2007).
	Complex Homoeopathy	*Complex homeopathy, also known as combination homeopathy or combination remedies, refers to a form of homeopathic treatment where multiple homeopathic remedies are combined into a single preparation. It can be used as a complementary approach in the treatment of depression, but it's important to note that the evidence supporting its effectiveness is limited and mixed* (Thachil, 2007).	Another RCT studied the effectiveness of Neurapas balance, a complex homoeopathic remedy, which is a combination of St. John's wort, passion flower and valerian extracts, in 67 adult patients with mild depressive disorders according to ICD‐10 (Thachil, 2007).
	Relaxation training	*Relaxation training refers to a set of techniques and practices that aim to induce a state of relaxation in the body and mind. These techniques are often used to manage stress, promote well‐being, and improve overall mental and physical health* (Fung, 2012).	Another RCT with a small sample (37) of patients with DSM‐IV moderate depression, compared relaxation training, cognitive‐behavioral therapy and tricyclic antidepressants. This produced evidence in favour of relaxation training, with 73% of the relaxation group improving to set BDI criteria post‐treatment, compared to 82% for CBT and 29% for tricyclics (Thachil, 2007).

### 4.2 EGM framework: Indicators/outcomes

Table 2 lists outcomes, definitions, and examples. This study focuses on the scope the impact of non‐pharmacological interventions for older people with a diagnosis of depression. The outcome of interest for this EGM is the effectiveness and cost‐effectiveness of interventions. The most common health outcomes encompass various aspects, such as the change in depressive symptoms (including relief of the depressive mood and reduction of suicidal ideation), improvement in the quality of life, enhancement of physical fitness, and increase in social skills. In addition, health economic outcomes include cost‐effectiveness ratios, cost‐utility ratios, and incremental cost‐effectiveness ratios of different non‐pharmacological interventions.

**Table 2:** Categories, subcategories, definitions, and examples of outcomes.

Categories	Sub‐category	Sub‐category	Definition	Examples
Type of depression in older individual	Major depression	Major depressive disorder (MDD)	MDD, commonly known as clinical depression, is a mental health condition characterized by persistent feelings of sadness, loss of interest or pleasure in activities, and a variety of physical and cognitive symptoms. It is a significant and debilitating condition that affects a person's daily life, relationships, and overall well‐being (Belmaker, 2008).	“Our review supports the existing research literature on PST suggesting that it is an effective treatment for older people with MDD.” (Kirkham, 2016)
	Non‐major/sub‐threshold depression	Minor depression	Minor depression is defined as presence of clinically significant depressive symptoms which do not meet the threshold duration criterion or the number of symptoms necessary for the diagnosis of Major depressive disorder (MDD) as per the current nosology (Avasthi, 2018).	“The purpose of this study was to conduct a pilot clinical trial to test the feasibility and efficacy of an exercise program and anti‐depressant treatment compared with usual care in improving the emotional and physical functioning of older adults with minor depression.” (Brenes, 2007)
		Dysthymia: Persistent Depressive Disorder (PDD)	PDD, also known as dysthymia, is a chronic form of depression characterized by persistent feelings of sadness and a lack of interest or pleasure in daily activities. Unlike Major Depressive Disorder (MDD), which typically involves intense episodes of depression that come and go, PDD is a more long‐lasting condition (Klein, 2013).	To compare the effectiveness of pharmacotherapy and psychotherapy in primary care settings among older persons with minor depression or dysthymia (Williams, 2000).
		Adjustment disorder with depressed mood	Adjustment disorder is a psychological condition that occurs in response to a significant life stressor or event. In the case of adjustment disorder with depressed mood, the primary symptom is a depressed mood that is in excess of what would be expected given the nature of the stressor (Avasthi, 2018).	/
		Mixed anxiety and depressive disorder (MADD)	MADD is a diagnostic category that encompasses individuals who experience symptoms of both anxiety and depression, without meeting the full criteria for either disorder separately. It is recognized as a subtype of other specified anxiety and depressive disorders in the Diagnostic and Statistical Manual of Mental Disorders, Fifth Edition (DSM‐5) (Avasthi, 2018)	“In conclusion, group cognitive behavioural therapy is efficacious in reducing comorbid anxiety and depression in geriatric populations and gains maintain for at least three months.” (Wuthrich, 2013)
		Bipolar depression	Bipolar depression is a specific form of depression that occurs within the context of bipolar disorder, which is characterized by alternating periods of depression and mania or hypomania. Bipolar disorder is a chronic mental health condition that involves extreme mood swings and can significantly impact a person's daily life (Baldessarini, 2010).	“Electroconvulsive therapy for bipolar depression was associated with very high response rates. The strongest prognostic factors were higher age, absence of comorbid obsessive‐compulsive disorder or personality disorder, and less prior pharmacologic treatment.” (Popiolek, 2019)
		Depressive Disorder with Another Medical Condition	Depressive Disorder with Another Medical Condition, also known as comorbid depression, refers to the presence of both a depressive disorder and a coexisting medical condition. It is common for individuals with chronic medical illnesses or conditions to experience symptoms of depression alongside their physical health challenges (Sylvia, 2015).	“This brief review describes the unique etiologies, features, and treatments for depressive syndromes among older adults in the oncology setting, drawing on the literature and prevailing depression management guidelines from both psycho‐oncology and geriatric depression research.” (Saracino, 2019)
		Unspecified depressive symptom	/	/
		Other specific depressive symptoms	/	/
Change in depressive symptoms for older individuals	Persistent sadness	A persistent feeling of sadness, emptiness, or anxious that lasts for an extended period (Baba, 2022).	“T‐CBT outperformed TAU on all depression, anxiety, and quality of life measures…. Improvements were moderated by a reduction in negative thoughts in the T‐CBT group only, reflecting treatment target engagement.” (Dobkin, 2020)	
	Loss of interest and pleasure	Loss of interest and pleasure is a common symptom associated with depression. It refers to a diminished ability to derive enjoyment or satisfaction from activities that were once pleasurable (Baba, 2022).	“Major depression is characterized by depressed mood and diminished interest or pleasure in all, or almost all, activities.” (Ontario, 2019)	
	Changes in appetite	Significant changes in appetite, such as a loss of appetite or increased appetite. These changes may result in unintentional weight loss or weight gain (NIH).		
	Sleep disturbance	Sleep disturbances refer to disruptions in the normal sleep pattern, leading to difficulties falling asleep, staying asleep, or experiencing restful sleep (Paudel, 2013)	“The current archival analyses examine the direct and indirect effects of cognitive behavioral therapy for insomnia (CBT‐I) on depression in cancer survivors.” (Peoples, 2019)	
	Fatigue or Loss of energy	Feeling constantly tired, lacking energy, and experiencing a decrease in motivation and physical stamina (NIH).		
	Feelings of worthlessness or guilt	Negative beliefs about oneself, feelings of worthlessness, excessive guilt, or self‐blame (NIH).		
	Cognitive changes	Difficulties with concentration, memory, decision‐making, or slower thinking. These cognitive changes can sometimes be mistaken for age‐related cognitive decline or other cognitive disorders (NIH).	The secondary outcomes assessed by the reviewed studies included quality of life, rumination and cognitive functioning (Apóstolo, 2016).	
	Psychomotor changes	Observable changes in physical movement, such as slowed movements, restlessness, or agitation (NIH).		
	Withdrawal or social isolation	Withdrawing from social activities, reducing social interactions, or experiencing a sense of loneliness or isolation (NIH).		
	Physical symptoms	Older adults may more commonly express their depressive symptoms through physical complaints, such as persistent pain, gastrointestinal distress, headaches, or other somatic symptoms (NIH).	Multisite pain, pain severity and frequency were the best predictors of late life depression (Denkinger, 2014).	
	Suicide intention	Suicide intention is the thought or act of intentionally causing one's own death (Szanto, 2001)	“Because the scientific literature on psychosocial suicide prevention interventions in the elderly is still scant, we conducted a mini‐review to take stock of the situation. “ (Zeppegno, 2019)	
Life and social	Quality of life	Quality of life refers to an individual's overall well‐being and satisfaction with various aspects of their life. It encompasses both objective and subjective factors that contribute to a person's sense of fulfillment, happiness, and overall functioning (Fallowfield, 2009).	“This study was aimed to investigate in a sample of Spanish elderly whether measures of physical activity are related to health‐related quality of life (HRQoL) and symptoms of depression in community dwelling and institutionalized elderly.” (Salguero, 2011)	
	Physical fitness	Physical fitness refers to the state of being in good physical condition, characterized by strength, endurance, flexibility, and overall health (Wilder, 2006).	“The review of the literature and the meta‐analysis demonstrated a relationship between low muscle strength and intensified depressive symptoms in older populations. Bearing in mind that depression is often unrecognized or underdiagnosed among older patients, lowered muscle strength should be an important sign for physicians and an incentive to screen them for depression.” (Zasadzka, 2021)	
	Social skill	Social skills refer to the abilities and competencies that enable individuals to interact effectively and appropriately with others in various social situations (Tse, 2004).	“Psychotherapy for depression results in small to moderate improvements in social functioning. These improvements are strongly associated with, but not fully explained by, improvements in depressive symptoms.” (Renner, 2014)	
Adverse events		Adverse events are closely monitored and reported during clinical trials and research studies. They encompass any unfavorable or unintended medical occurrences experienced by participants, whether or not they are related to the intervention being investigated (Vincent, 2003).	“No information about deterioration, adverse events, or any other harmful effects was presented in any of the included trials. No trial indicated that such effects had been monitored. The quality of evidence for safety was therefore graded as very low for all interventions.” (Jonsson, 2016)	
Cost‐effectiveness results	Incremental cost‐effectiveness ratios (ICERs)	An incremental cost‐effectiveness ratio is a summary measure representing the economic value of an intervention, compared with an alternative (comparator). It is usually the main output or result of an economic evaluation. An ICER is calculated by dividing the difference in total costs (incremental cost) by the difference in the chosen measure of health outcome or effect (incremental effect) to provide a ratio of ‘extra cost per extra unit of health effect’ – for the more expensive therapy vs the alternative (HERC)	The intervention resulted in 1,072 QALYs gained with the corresponding ICER being dominant (Engel, 2021).	
	Cost‐effectiveness ratios	Cost‐effectiveness analysis is a tool used to aid decisions about which medical care should be offered. It is a method of comparing the cost and effectiveness of two or more alternatives. Such comparisons are useful when one of the alternatives being considered is standard care, as this allows the decision maker to consider whether an innovation is better than the status quo (HERC).	The aim of the study was to evaluate the cost‐effectiveness of the Friendship Enrichment Programme (FEP) and a volunteer‐led internet and computer training (VICT) intervention to reduce loneliness in older adults and, in turn, prevent depression (Engel, 2021).	
	Cost‐utility ratio	Cost‐utility analysis (CUA) is one type of economic evaluation that can help you compare the costs and effects of alternative interventions. CUA measures health effects in terms of both quantity (life years) and quality of life. These are combined into a single measure of health: quality‐adjusted life years (QALYs) (GOV.UK).	Cost‐utility analysis (CUA) is a method to support decisions on efficient allocation of resources in health policy. The objective of our study was to systematically review CUA of CBT in the treatment of patients suffering from MDD (Brettschneider, 2015).	

### 4.3 EGM framework: Population dimension

The primary population of interest for this map includes older adults (aged 60 years and above) with a diagnosis of depression. We will further categorize the population based on sex (female, male), health state (depression alone, depression with physical disease(s), depression with other psychological disorder(s)), and the type of depression. The population dimension will be listed as a filter.

## 5 INCLUSION AND EXCLUSION CRITERIA

### 5.1 Types of study designs

The study designs eligible for inclusion in this EGM are:

- RCTs on the effectiveness of interventions that utilize various forms of control groups.
- Economic evaluations (EEs, e.g., cost‐effectiveness studies and cost‐utility studies) of interventions.
- And Systematic reviews (SRs), including original studies conducted as RCTs or quasi‐experimental studies to evaluate the effectiveness and cost‐effectiveness of interventions.

Trial registries and protocols for ongoing research will also be included. Additionally, qualitative studies, integrative reviews, rapid reviews, reviews of reviews, and evidence synthesis/summaries will also be excluded.

### 5.2 Types of interventions/problems

We will include comparisons of non‐pharmacological interventions with other non‐pharmacological interventions, pharmacological interventions, or usual care. We will exclude interventions that do not meet the definition of a non‐pharmacological treatment.

### 5.3 Types of populations

The population of relevance to this EGM consists of older adults with a diagnosis of depression. According to the older age classification by the WHO, older adults are defined as 60 years and older. Depression will be diagnosed according to the Diagnostic and Statistical Manual of Mental Disorders, 3rd edition (DSM‐III) (APA, 1980), the DSM, 3rd revised edition (DSM‐III‐R) (APA, 1987), the DSM, 4th edition (DSM‐IV) (APA, 1994), the DSM, 4th text revised edition (DSM‐IV‐TR) (APA, 2000), or the DSM, 5th edition (DSM‐5) (APA, 2013), the International Classification of Diseases, Tenth Revision (ICD‐10) (WHO, 1992), Research Diagnostic Criteria (RDC) (Spitzer, 1978), Geriatric Mental State (GMS) (Gurland, 1976), or as defined by trialists.

### 5.4 Types of outcome measures

We will include primary studies and systematic reviews that assess the effectiveness and cost‐effectiveness of non‐pharmacological interventions in addressing various aspects among older people with a diagnosis of depression. These aspects include depressive symptoms, quality of life, physical fitness, and social skills, adverse events, as well as health economic outcomes. Outcomes will be extracted and presented as described in the included articles.

### 5.5 Types of settings

We will include interventions in any setting, that is, participants treated in a range of settings (hospitals, community, and care homes). We will code the settings so that the evidence can be filtered according to settings.

### 5.6 Search methods and sources

This EGM will include relevant RCTs, economic evaluations, and systematic reviews, including both published and ongoing research. To achieve this, specific Medical Subject Headings (MeSH) and free text terms such as “non‐pharmacological interventions” will be combined with terms for “depressive disorder.” These terms will be combined with terms for publication type, keywords such as “randomized controlled trial,” “systematic review,” “cost‐effectiveness analysis,” and others, using Boolean logic operators (and, or).

A draft search strategy for MEDLINE via Ovid SP will be designed, as outlined in Table 3. The strategy will be shared with the review team and the Advisory Board for feedback and relevant revisions will be implemented. The final search strategy and search resources will be discussed and agreed upon within the review team.

**Table 3:** Search strategies.

Database	Search strategies
MEDLINE via Ovid SP	#1 exp Depression/
	#2 exp Depressive Disorder/
	#3 exp Depressive Disorder, Major/
	#4 exp Mental Disorders/
	#5 exp Mental Health/
	#6 exp Dysthymic Disorder/
	#7 exp Bipolar Disorder/
	#8 exp Adjustment Disorders/
	#9 exp Seasonal Affective Disorder/
	#10 exp Psychological Distress/
	#11 exp Long‐Term Synaptic Depression/
	#12 (depress* or distress* or dysthymi* or “affective disorder*” or “affective symptom*” or melanchol* or “mood disorder*” or “adjustment disorder*” or “mental health” or “mental disorder*”).ab,kw, ti.
	#13 (#1 or #2 or #3 or #4 or #5 or #6 or #7 or #8 or #9 or #10 or #11 or #12)
	#14 exp Aged/
	#15 exp “Aged, 80 and over”/
	#16 (elder* or geriatri* or senil* or older or “old age” or “late life” or octogenarian or nonagenarian).ab, kw,ti.
	#17 (#14 or #15 or #16)
	#18 #13 and #17
	#19 exp Psychosocial Intervention/
	#20 exp Animal Assisted Therapy/
	#21 exp Art Therapy/
	#22 exp Cognitive Behavioral Therapy/
	#23 exp Therapy, Computer‐Assisted/
	#24 exp Emotion‐Focused Therapy/
	#25 exp Music Therapy/or exp Dance Therapy/
	#26 exp Behavior Therapy/
	#27 exp Dialectical Behavior Therapy/
	#28 exp Psychotherapy, Psychodynamic/
	#29 exp Problem Solving/
	#30 exp Mindfulness/
	#31 exp Family Therapy/
	#32 exp Electroconvulsive Therapy/or exp Biofeedback, Psychology/or exp Transcranial Magnetic Stimulation/
	#33 exp Exercise Therapy/
	#34 exp Medicine, Chinese Traditional/or exp Homeopathy/or exp Acupuncture/or exp Acupuncture Therapy/
	#35 (“non‐pharmacological treatment*“ or “non‐pharmacological intervention*” or “psychosocial intervention*” or “acceptance and commitment therap*” or ACT or “animal‐assisted therap*” or “art therap*” or “behavioral therap*” or BT or “cognitive behavioral therap*” or CBT or “cognitive bias modification” or CBM or “computer‐assisted therap*” or “dance and movement therap*” or DMT or “dialectical behaviour therap*” or DBT or “emotion‐focused therap*” or EFT or “Metacognitive therap*“ or MCT or “Problem solving therap*” or PST or “Psychodynamic therap*“ or “Solution‐focused therap*” or SFT or “Humanistic therap*” or Mindfulness or “Metacognitive therap*” or MCT or “family therap*” or “electroconvulsive therap*” or ECT or “Repetitive transcranial magnetic stimulation” or rTMS or “neurosurgical intervention*” or biofeedback or “exercise” or “Traditional Chinese medicine” or TCM or acupuncture or homeopathy or “complementary and alternative medicine intervention*” or CAM).ab,kw,ti.
	#36 (#19 or #20 or #21 or #22 or #23 or #24 or #25 or #26 or #27 or #28 or #29 or #30 or #31 or #32 or #33 or #34 or #35)
	#37 randomized controlled trial.pt.
	#38 controlled clinical trial.pt.
	#39 randomized.ab.
	#40 placebo.ab
	#41 clinical trials as topic.sh.
	#42 randomly.ab.
	#43 trial.ti.
	#44 (#37 or #38 or #39 or #40 or #41 or #42 or #43)
	#45 Systematic Review.pt.
	#46 exp Systematic Review as Topic/
	#47 Meta‐Analysis.pt.
	#48 exp Meta‐Analysis as Topic/
	#49 (“systematic review” or meta‐analysis or meta).ti.
	#50 (#45 or #46 or #47 or #48 or #49)
	#51 exp Budgets/or exp “Costs and Cost Analysis”/or Economics/or exp Economics, Hospital/or exp Economics, Medical/or Economics, Nursing/or exp “Fees and Charges”/or exp Cost‐Benefit Analysis/
	#52 (cost* adj2 (effective* or utilit* or benefit* or minimi*)).ab,kw,ti.
	#53 (cost* or economic*).ti. or (budget* or fee* or financ* or price* or pricing or resourc* allocat*).ti,ab.
	#54 (#51 or #529 or #53)
	#55 (#44 or #50 or #54)
	#56 #18 and #36 and #55

A wide range of bibliographic databases and websites will be searched to cover all relevant studies. The primary list of databases is presented as follows:

- MEDLINE via Ovid SP
- PubMed (excluding MEDLINE)
- CINAHL via EBSCOhost
- PsycINFO (EBSCO)
- Cochrane Central Register of Controlled Trials (CENTRAL), via the Cochrane Register of Studies Online
- (CRSO)/or via the Cochrane Library
- AgeINFO (ageinfo.cpa.org.uk)
- Epistemonikos (www.epistemonikos.org)
- The Cochrane Library (www.cochranelibrary.com)
- The Campbell Library (www.campbellcollaboration.org/ better‐evidence.html)
- 3ie Systematic Review Database (www.3ieimpact.org/evidence-hub/publications/systematic-reviews)
- US National Institutes of Health Ongoing Trials Register ClinicalTrials. gov (www.clinicaltrials.gov)
- World Health Organization International Clinical Trials Registry Platform (ICTRP) (https://www.who.int/clinical-trials-registry-platform)

### 5.7 Searching other resources

We will search various sources of gray literature and the websites of organizations conducting mental health research to identify relevant unpublished studies. The following resources and websites will be consulted for this purpose:

- American Psychological Association (www.apa.org)
- Centers for Disease Control and Prevention (www.cdc.gov)
- National Institute of Mental Health (www.nimh.nih.gov)
- World Health Organization (www.who.int)
- Open Grey (www.opengrey.eu)

We will also search Google Scholar (Scholar.google.com) by keywords and retrieve the results of the first 20 pages. Additionally, we will search the reference lists of all identified studies, relevant review articles, and current treatment guidelines to identify other potentially eligible studies.

The search will be executed and updated as necessary before publication. We will provide the actual data of the electronic search at the review stage.

## 6 ANALYSIS AND PRESENTATION

### 6.1 Report structure

The EGM report will include the following sections: executive summary, background, methods, results, and conclusion.

The executive summary will summarize the report, highlighting the main findings and their future implications for policy and research. The background section will provide a description of the challenges older individuals with depression face in non‐pharmacological interventions. This section will also outline the objectives of the EGM and describe its scope by defining the intervention and outcomes framework.

The methods section will outline the data sources, methods for searching, inclusion and exclusion criteria, screening, quality appraisal, and data extraction methods in detail. This section will also highlight the search strategy and the Preferred Reporting Items for Systematic reviews and Meta‐Analyses (PRISMA) (Page, 2021). Further, the full search strategy will be reported, including details on restrictions and filters.

The results section will present the number, type, and quality of studies retrieved for the intervention and outcome categories. This section will also include the interactive EGM, thus providing a graphical representation of the available evidence.

Finally, in the concluding section of the report, we will discuss the implications of the EGM for researchers, decision‐makers, and other important stakeholders, and we will identify key areas to be noted for future research.

We will present any changes made between the protocol and the final report.

In addition, the report will include the following tables and figures may include:

- Figure: PRISMA diagram
- Figure: EGM—primary studies (non‐interactive)
- Figure: EGM—systematic reviews (non‐interactive)
- Figure: online interactive EGM—with filters
- Table: Number of studies by intervention and subcategories
- Table: Number of studies by population
- Table: Number of studies by intervention category and study confidence

### 6.2 Filters for presentation

The results of the online interactive EGM will be presented as a matrix of intervention categories (or subcategories) (on the y‐axis) and outcomes (on the x‐axis). We will use bubbles of varying sizes to present the studies included. Different colors will be used for different types of study designs (i.e., SR and primary studies). We will additionally employ the following filters:

- Populations information: sex (female, male); health state (depression alone, depression with physical disease(s), depression with other psychical disorder(s))
- Country income group: low‐income, lower‐middle‐income, upper‐middle‐income, high‐income (defined according to the World Bank country classification 2022)
- Study setting: hospital, community, and care homes
- Study design: primary studies and SRs of the effectiveness of interventions; primary studies and SRs of the economic evaluations of interventions
- Study status: protocol, completed, ongoing
- Intervention type: psychological interventions, non‐pharmacological medical interventions, physical interventions, and CAM interventions
- Depressive symptoms: major depression, non‐major/sub‐threshold depression
- Quality of studies: critical low, low quality, middle quality, and high quality
- Conflict of interest: yes, no, or unclear
- Funding: yes, no, or unclear

### 6.3 Dependency

The unit of analysis for this EGM will be the included studies (i.e., SRs and primary studies of effectiveness and cost‐effectiveness). If multiple papers are published from the same study, the most recent open‐access publication will be included in the EGM. If previous publications from the same study include different outcome measures, these papers will only be included to report on missing outcomes. If this situation arises, all publications from the same study will be treated as one single study. It is likely that SRs will include the RCTs in the map, and that more than one SR will include the same RCT. All relevant RCTs will be included, whether they are included in an SR or not. The final EGM will identify the number of studies covered by the map and clearly list studies where multiple papers are published from the same study within the references.

### 6.4 Data collection and analysis

#### 6.4.1 Screening and study selection

All titles and abstracts will be screened by two independent authors (GK and YMY) based on intervention, study design, and population to identify eligible studies. The outcome will not be included in this process. We will obtain the full text of all potentially relevant reports/publications and two review authors (GK and YMY) will independently screen them and identify studies to be included, and the reasons for the exclusion of ineligible studies will be identified and documented. Any disagreements regarding inclusion will be addressed through discussion between the two authors or (if needed) adjudication by a third author (SWR). We will present a PRISMA flow diagram to show the process of study selection (Page, 2021).

### 6.5 Data extraction and management

A standardized data extraction and coding form will be used to extract descriptive data from all studies included in the map. Two independent researchers (SWR and GK) will independently code papers using the EPPI mapper (EPPI‐Mapper 2021). The extraction will be supervised by a third review author (WLL). Coding categories will be based on the intervention and outcome framework. Information on additional filters, such as population information, country income groups, study design, study setting, intervention type, effectiveness of interventions, cost‐effectiveness of interventions, depressive symptoms, quality of studies, conflict of interest, and funding will also be included.

### 6.6 Tools for assessing risk of bias/study quality of included reviews

All primary studies and systematic reviews will be assessed for risks of bias, quality, or confidence using the most appropriate tools. Systematic reviews will be appraised using AMSTAR‐2 (Shea, 2017). Primary studies of effectiveness will be appraised using the Cochrane Risk of Bias 2 tool (Sterne, 2019). Primary studies of cost‐effectiveness will be appraised using the Consolidated Health Economic Evaluation Reporting Standards 2022 (CHEERS 2022) (Husereau, 2022). These assessments will be completed independently by two authors (SWR and GK), and any conflicts will be resolved by consensus. In case of disagreements that cannot be reconciled between the two reviewers, a third reviewer will make the final decision (YKH).

### 6.7 Methods for mapping

For the development of the EGM, we will use the EPPI mapper (EPPI‐Mapper 2021) to screen the literature, extract data, and generate the visual interactive EGM.

## CONTRIBUTIONS OF AUTHORS

- Content: Wenru Shang, Liping Guo, Lili Wei, Junqiang Niu, Kehu Yang, Qian Wei.
- EGM methods: Wenru Shang, Liping Guo, Yanfei Li, Zheng Xu.
- Statistical analysis: Wenru Shang, Yujia Liu.
- Information retrieval: Wenru Shang, Yujia Liu, Ke Guo, Mingyan Yang.

## DECLARATIONS OF INTEREST

All authors have no conflicts of interest.

## PLANS FOR UPDATING THE EGM

Once completed, the EGM will be updated every 5 years, depending on funding. The lead author and co‐authors will be responsible for updating the EGM.

## SOURCES OF SUPPORT

### Internal sources

- Natural Science Foundation of Gansu Province (Project No. 22JR5RA510), China
- Fundamental Research Funds for the Major Project of the National Social Science Fund of China (Project No. 19ZDA142), China

### External sources

- None, China

## Supporting information

Supporting information.
