# Supplementary material for: PROTOCOL: Non‐pharmacological interventions for older people with a diagnosis of depression: An evidence and gap map
Source: Campbell Syst Rev. 2023 Sep 27;19(4):e1354. doi: 10.1002/cl2.1354 (PMC10523358; doi:10.1002/cl2.1354)
Supplement: Supplementary file 1 — Supporting information. [file CL2-19-e1354-s001.docx]

**Response letter**

Dear Jo Thompson Coon,

Thank you for your letter and comments concerning our manuscript entitled “Non-pharmacological interventions for older people with a diagnosis of depression: An evidence and gap map” (ID: cl2.20220099.R1). Those comments are all valuable and helpful for revising and improving our paper. We have studied comments carefully and have made correction which we hope meet with approval. Revised portion are marked in the manuscript (REVMAN WEB). The main corrections in the paper and the responds to comments are as flowing：

1. Please clarify eligible study designs. In the objectives you state that you will map RCTs, economic evaluations and systematic reviews. In the Inclusion criteria, you state that you will include RCTs and evaluative quasi-experimental studies, economic evaluations and systematic reviews.

**Response**: Thanks for spotting this mistake. We have updated the indicators criteria section, as following:

“ The study designs eligible for inclusion in this EGM are:

- RCTs on the effectiveness of interventions that utilize various forms of control groups.
- ...”

1. Please remove reference to ‘elderly’ throughout.

**Response**: Many thanks for your suggestion. We have made the necessary update by changing the term previously used to “older”.

1. Please re-phrase this sentence: This EGM will be able to retrieve and include relevant primary studies and systematic reviews, both published and ongoing.

**Response**: Many thanks for your suggestion. We have updated this sentence:

“ This EGM will include relevant RCTs, economic evaluations and systematic reviews, including both published and ongoing research.”

1. I’m not sure how the search strategy reduces the risk of publication or time bias. Please remove this sentence from the protocol.

**Response**: Many thanks for your comments. We have removed this sentence from the protocol.

1. There are some remaining typos and grammatical errors. For example, in the objectives you refer to an EMG rather than an EGM, in Figure 1 ‘Higher well-being’ might be better expressed as ‘Improved wellbeing’ and this sentence in the section EGM Framework indicators/outcomes ‘This study focuses on the scope the impact of non-pharmacological interventions for older people with a diagnosis of depression.’ doesn’t make sense. Please carefully check the grammar throughout and ask a native English speaker to read and edit the protocol before resubmission.

**Response**: Thanks for spotting this mistake. The protocol has been thoroughly revised and edited by native English speakers. Figure 1 also have been checked and revised. A certificate of English editing attached as follows. We hope it can meet the journal’s standard.


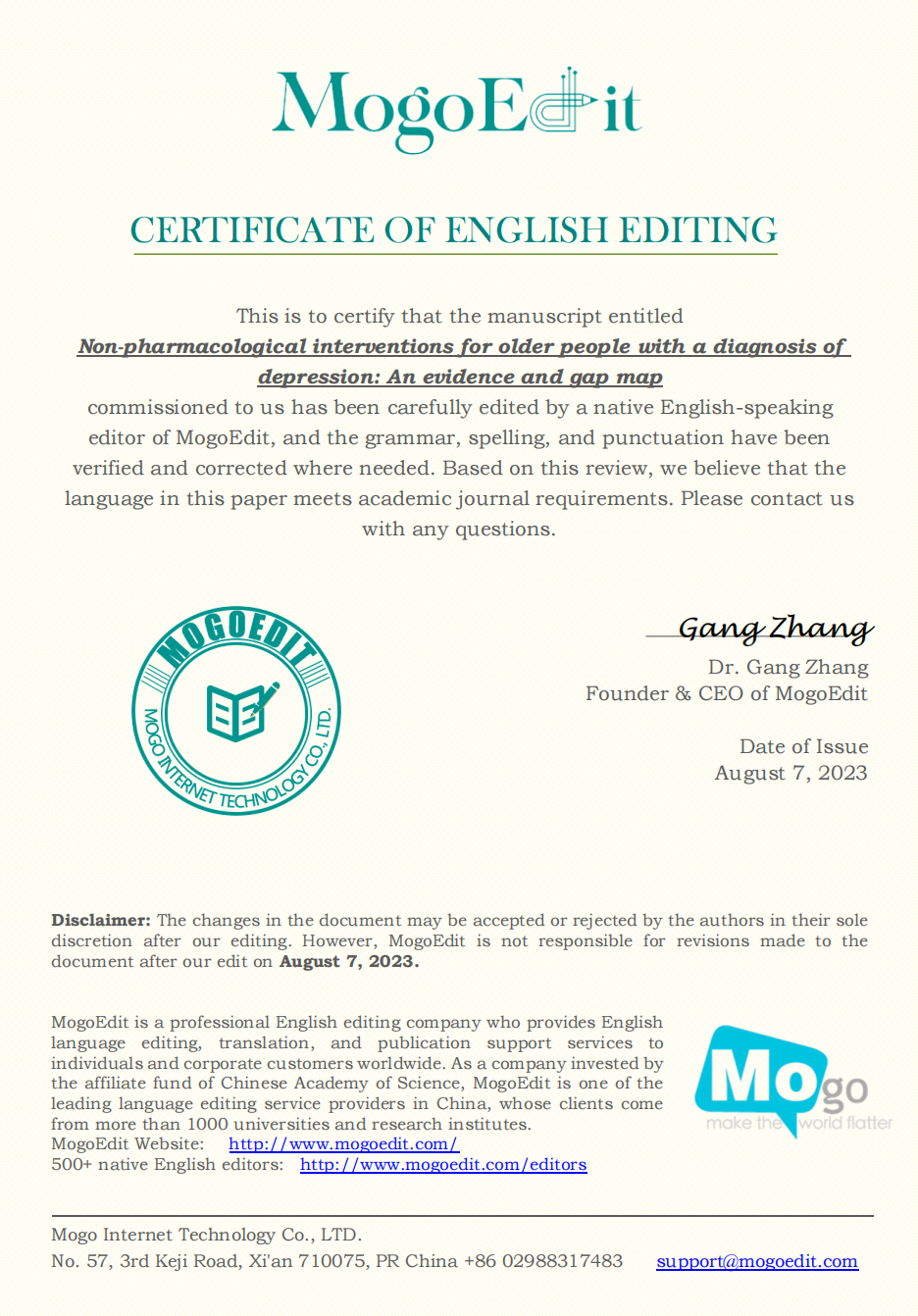


1. Please remove Tables 1 and 2 from the protocol.

**Response**: Many thanks for your comments. We have removed Tables 1 and 2 from the protocol.

1. Please check Table 5 for grammatical errors and replace the description of depression with a recognised definition, alternatively remove the table and include the definitions within the text.

**Response**: Many thanks for your comments. We have removed Table 5 and updated the section of EGM framework: population dimension, as following:

“The primary population of interest for this map includes older adults (aged 60 years and above) with a diagnosis of depression. We will further categorize the population based on sex (female, male), health state (depression alone, depression with physical disease(s), depression with other psychical disorder(s)), and the type of depression. The population dimension will be listed as a filter.”

We appreciate for your work earnestly, and hope that the correction will meet with approval.

Once again, thank you very much for your comments and suggestions.

Best regards,

Wenru Shang

1. *WHO Collaborating Center for Guideline Implementation and Knowledge Translation, Lanzhou University, Lanzhou, China;*

*2. Evidence Based Medicine Center, School of Basic Medical Sciences, Lanzhou University, Lanzhou, China*

*Address:No.222, TianShui Road(south), Lanzhou, P. R. China*

*Zip code:730000*

*Email:shangwr@lzu.edu.cn*
